# Attachment representations in high intellectual potential (HIP) children compared to non-HIP children during development

**DOI:** 10.1192/j.eurpsy.2024.917

**Published:** 2024-08-27

**Authors:** S. Hamdioui, L. Vaivre-Douret

**Affiliations:** ^1^Faculty of Medicine, University of Paris-Saclay, UVSQ, INSERM Unit 1018-CESP, Villejuif; ^2^Faculty of Health, Department of Medicine, University of Paris Cité; ^3^Chair of Neurodevelopmental Clinical Phenotyping, Institut Universitaire de France (IUF); ^4^ Necker Enfants Malades University hospital, AP-HP. Centre; ^5^Department of Endocrinology, Necker-Enfants Malades Hospital, IMAGINE Institute, Paris, France

## Abstract

**Introduction:**

The studies about developmental bases of attachment in healthy children with high intellectual potential (HIP) are rare.Moreover, the literature underline socio-emotional disorders in HIP and difficulties with behavioral adjustment of parents.

**Objectives:**

We aimed to explore the developmental trajectory of attachment in HIP children without psychological or learning disorders.

**Methods:**

The french version of the Adolescent-Unresolved-Attachment-Questionnaire (QANRA: internal consistency=0.74-0.82; test-retest =0.58-0.83) was analyzed in 80 healthy children (50 HIP with IQ>130 vs. 30 non-HIP), aged 7-to-13-years-old (mean 10y; SD 1.8). All children were recruited in private and public schools in Paris.

**Results:**

There was no significant difference between the groups. However, when we looked at the developmental trajectory by distinguishing the period of adolescence [7-10 years (56% in the HIP group vs. 53% in the non-HIP); 11-13 years (44% in the HIP group vs. 47% in the non-HIP)], we have noted a significantly early integration of resolved attachment in the HIP children that seems to remain stable in adolescence.

**Conclusions:**

Our findings highlight the early onset of attachment with a harmony of intellectual/psycho-affective development in HIP children without skipping stages, but more quickly and effectively. This could potentially be explained by their cognitive abilities, particularly the theory of mind and the executive functions, known to be significantly more efficient in HIP children without neurodevelopmental disorders.

**Disclosure of Interest:**

None Declared

